# Using green fluorescent malaria parasites to screen for permissive vector mosquitoes

**DOI:** 10.1186/1475-2875-5-23

**Published:** 2006-03-28

**Authors:** Friedrich Frischknecht, Beatrice Martin, Isabelle Thiery, Catherine Bourgouin, Robert Menard

**Affiliations:** 1 Unité de Biologie et Génétique du Paludisme, Institut Pasteur, 25-28 Rue du Dr Roux, 75015 Paris, France; 2 Centre de Production et d'Infection des Anopheles, Institut Pasteur, 25-28 Rue du Dr Roux, 75015 Paris, France; 3 Department of Parasitology, University of Heidelberg Medical School, Tel 49-6221-566537, Fax 49-6221-564643, Im Neuenheimer Feld 324, 69120 Heidelberg, Germany; 4 CNRS, Institut Alfred Fessard, Neurobiologie Génétique et Intégrative, Avenue de la Terrasse, 91198 Gif-sur-Yvette, France

## Abstract

**Background:**

The *Plasmodium *species that infect rodents, particularly *Plasmodium berghei *and *Plasmodium yoelii*, are useful to investigate host-parasite interactions. The mosquito species that act as vectors of human plasmodia in South East Asia, Africa and South America show different susceptibilities to infection by rodent *Plasmodium *species. *P. berghei *and *P. yoelii *infect both *Anopheles gambiae *and *Anopheles stephensi*, which are found mainly in Africa and Asia, respectively. However, it was reported that *P. yoelii *can infect the South American mosquito, *Anopheles albimanus*, while *P. berghei *cannot.

**Methods:**

*P. berghei *lines that express the green fluorescent protein were used to screen for mosquitoes that are susceptible to infection by *P. berghei*. Live mosquitoes were examined and screened for the presence of a fluorescent signal in the abdomen. Infected mosquitoes were then examined by time-lapse microscopy to reveal the dynamic behaviour of sporozoites in haemolymph and extracted salivary glands.

**Results:**

A single fluorescent oocyst can be detected in live mosquitoes and *P. berghei *can infect *A. albimanus*. As in other mosquitoes, *P. berghei *sporozoites can float through the haemolymph and invade *A. albimanus *salivary glands and they are infectious in mice after subcutaneous injection.

**Conclusion:**

Fluorescent *Plasmodium *parasites can be used to rapidly screen susceptible mosquitoes. These results open the way to develop a laboratory model in countries where importation of *A. gambiae *and *A. stephensi *is not allowed.

## Background

*Plasmodium berghei *is one of the most commonly studied *Plasmodium *species, particularly for elucidating the interactions between the parasites and their hosts [1-5]. The natural mammalian host of *P. berghei *is the African tree rat *Grammomys surdaster *and its natural mosquito vector is *Anopheles dureni *[6,7]. *P. berghei *is a species of choice for studies employing genetic manipulations due to the relative ease of parasite transfection and the function of many parasite genes has already been investigated in this species [8]. Recently, a method has been developed in *P. berghei *that will now permit the inactivation of essential genes specifically at pre-erythrocytic stages of the parasite [9]. Furthermore, the use of fluorescently labeled parasites has given unprecedented insights into the behaviour of these parasites within living insects and mice [10-13]. Although there are differences between the rodent and human-infecting *Plasmodium *parasites at the genomic, antigenic and cellular level [14,15], it is nonetheless clear that the rodent parasites are useful for elucidating the molecular basis of the core biology of *Plasmodium*, which often cannot be addressed with human parasites.

*Plasmodium spp *that infect humans are transmitted by a range of different mosquito species. While one of the main malaria vectors in Asia is *Anopheles stephensi*, the main vector in Africa is *Anopheles gambiae*. Both mosquito species are commonly used in laboratory experiments to study host-parasite interactions [16]. Far less common are studies using the main South American malaria vector, *Anopheles albimanus *[1]. This might be partly due to the facts that *P. berghei *has been reported to not infect *A. albimanus *[17] and that *Plasmodium yoelii *sporozoites generated in *A. albimanus *have been described as being non-infectious to the rodent host [18]. Since *A. gambiae, A. stephensi *and *A. albimanus *are all amenable to genetic modification [19-21], an *A. albimanus *– *P. berghei *system would be an interesting addition to the existing laboratory models. In addition, establishment of such a model, or similar models for other parasite species, would be valuable for host-vector-parasite interaction studies in countries where importation of *A. gambiae *and *A. stephensi *is not allowed. Here, using a fluorescent parasite line, *A. albimanus *mosquitoes were screened to check whether it was permissive for *P. berghei *development. It was found that *P. berghei *was able to infect *A. albimanus *and to develop into infectious sporozoites within this mosquito species.

## Methods

### Mosquito infection

For all infections, *P. berghei *(strain NK65 or ANKA) lines expressing the green fluorescent protein were used that allowed the detection of oocysts and sporozoites in living mosquitoes and dissected organs [11,22,29]. *A. stephensi *(strain Sda500), *A. gambiae *(strain Yaoundé) and *A. albimanus *(strain STECLA) mosquitoes were reared at the Center for Production and Infection of *Anopheles *(CEPIA) of the Institut Pasteur using standard procedures. Mosquitoes were fed on *P. berghei*-infected mice (parasitaemia >1%) 3–5 days (*A. stephensi *and *A. gambiae*) or 3–8 days (*A. albimanus*) after emergence, kept at elevated humidity (70% relative humidity) for up to 6 weeks in dedicated incubators or rooms at 21°C and feed on 10% (w/v) sucrose solution with or without supplements (see text) and received one additional non-infected blood meal after 1 or 2 weeks. The mosquitoes were allowed to lay their eggs on wet filter paper deposited in a plastic Petri dish.

### Observation of infected mosquitoes

**Figure 1:**
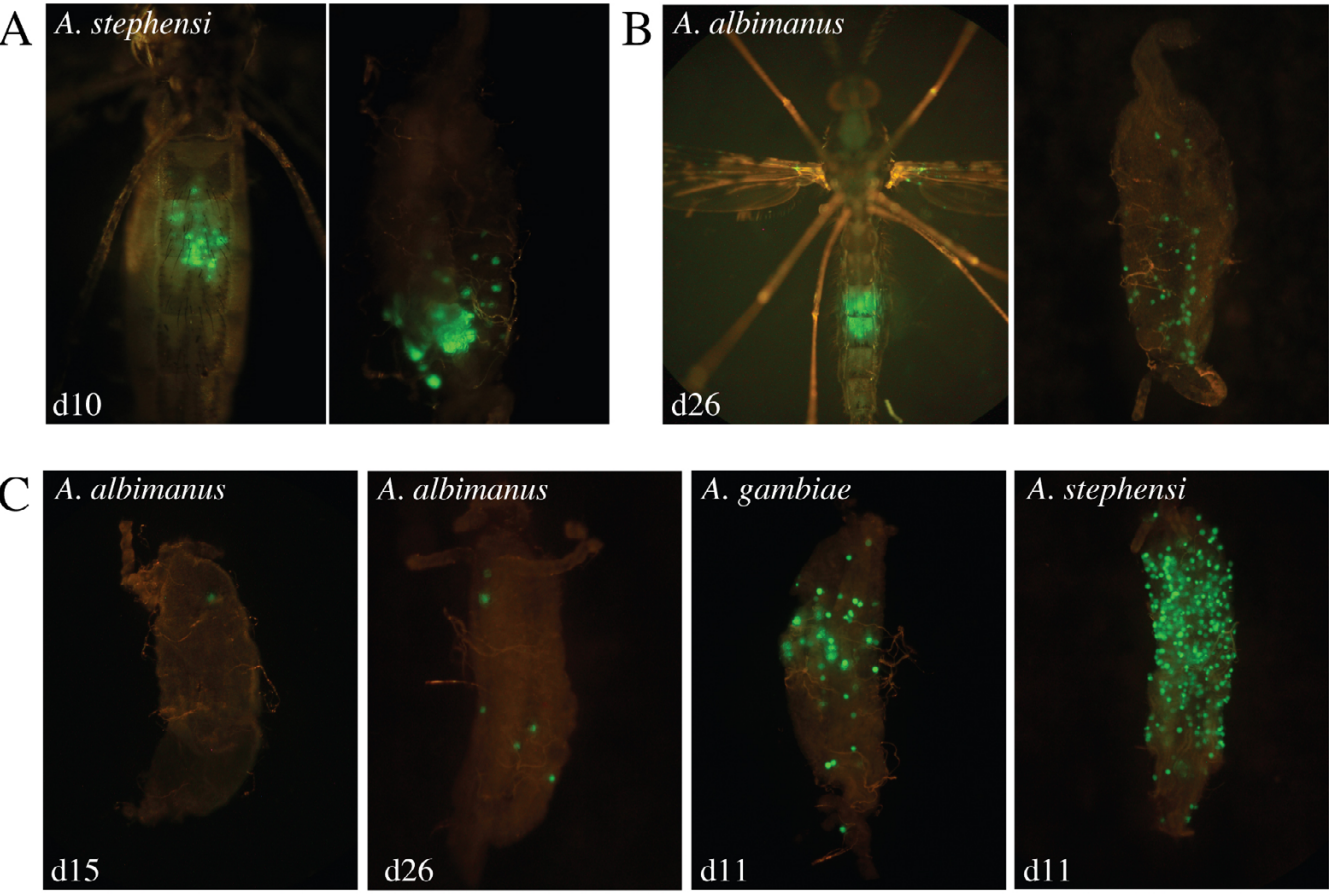
Detecting development of oocysts in *Anopheles *midguts. (A) Abdomen and dissected midgut of an infected *A. stephensi *mosquito with green fluorescent *P. berghei*. Note that single oocysts can be detected in the intact mosquito, while multiple oocysts give a blurred signal. (B) Oocyst derived fluorescence detected in a well infected living *A. albimanus *mosquito (left) and an isolated midgut (right) 26 days post infection. In the mosquito the fluorescence appears blurred due to the opaque nature of the abdomen's chitin. (C) Four representative photographs from midguts of infected *A. albimanus*, *A. gambiae *and *A. stephensi *mosquitoes. The days after the infectious blood meals are indicated.

Whole mosquitoes or isolated midguts were observed at 10–18 days (*A. stephensi *and *A. gambiae*) or 10–26 days (*A. albimanus*) after the infectious blood meal. Sporozoites were isolated from infected salivary glands 15–22 days (*A. stephensi*) and 15–35 days (*A. albimanus*) after the infectious blood meal. Infected salivary glands were dissected and kept on ice in phosphate buffered saline, or tissue culture medium (RPMI) with or without 5% foetal calf serum. While no difference in the movement of sporozoites in PBS or RPMI was observed, the addition of FCS stimulated motility in both media [11]. For screening, mosquitoes were anaesthetized on ice and investigated under a Nikon SMZ 1500 fluorescent stereomicroscope. For longer visual observations, mosquitoes were immobilized on glass slides with small droplets of super glue. Immobilized mosquitoes and isolated midguts in glass-bottom well dishes (Mattek, USA) were photographed on a Nikon SMZ 1500 fluorescent stereomicroscope with an attached Nikon Coolpix digital camera. For example, the images of Figure 1a were exposed for 0.5 seconds to reveal the fluorescence with a long pass FITC filter and the white light adjusted to appropriately illuminate the background. Depending on fluorescent intensity exposures were varied from 0.25 to 4 seconds. Infected mosquitoes, sporozoites within isolated salivary glands and isolated sporozoites were imaged as described previously [11]. Images and movies were analysed and processed with the Adobe software package and ImageJ.

### Infection of mice

All experiments using mice were approved by the committee of Institut Pasteur and were performed according to the applicable guidelines and regulations. For infection by mosquito bite or by intra-dermal injection, C57Bl/6 mice (Janvier, France) were anaesthetised with ketamine-xylazine. Rodents were injected in the food pad with 3 μl PBS containing sporozoites with a modified Hamilton micro-syringe (Precision Instruments, USA).

## Results and discussion

Mosquitoes were screened from day 10 after the infectious blood meal for the presence of fluorescent oocysts in the midgut. In *A. stephensi *and *A. gambiae *a fluorescent signal in the abdomen could readily be detected in intact mosquitoes (Figure 1a and data not shown). Dissection of the mosquitoes revealed that the fluorescence originating from even a single oocyst can be detected in intact mosquitoes. Detection was facilitated if the mosquitoes were given a blood meal prior to observation, which expanded the abdomen and thus decreased its opacity. In the vast majority of *A. albimanus*, there was no fluorescent signal. However, a small number of mosquitoes showed a weak fluorescence signal in their abdomen. To investigate if indeed a small number of *A. albimanus *were allowing the development of *P. berghei*, female mosquitoes were first observed intact and then dissected at various times (see methods) after the infecting blood meal. Less than 4% of *A. albimanus *were infected with one or more (maximum 30) oocysts, although typically all females in a cage had taken a potentially infectious blood meal (Table 1, Figure 1). In control experiments, between 50 and 95% of *A. stephensi *were infected with several dozen to over one hundred oocysts, and the salivary glands of infected *A. stephensi *usually contained over 10,000 sporozoites per gland.

**Table 1:** Evaluation of infected *A. albimanus *mosquitoes fed on sucrose solution with or without supplemented PABA and Pen/Strep. In total 14 infections were performed with mosquitoes fed on sugar (3 of these resulted in no infected midguts) and 20 infections with mosquitoes fed on PAPA+Pen/Strep. During each infection between 50 and 250 mosquitoes were investigated. Data shown are mean ± standard deviation.

	Sugar	Sugar with PABA+Pen/Strep
Infected mosquitoes Range, number of infections	3.9 ± 4.3% 0 – 16.6%, n = 14	19.2 ± 9.7% 4.4 – 44%, n = 20
% of infected midguts with < 5 oocysts	80.3 ± 25.3	49.5 ± 18.1
% of infected midguts with 5–20 oocysts	9.1 ± 16.8	28.1 ± 14.4
% of infected midguts with 20 – 50 oocysts	10.6 ± 23.9	7.3 ± 7.5
% of infected midguts with > 50 oocysts	0	5.3 ± 9.1
Mosquitoes with sporozoites in the haemolymph but no more oocysts in the midgut	0	9.4 ± 12.3%

**Figure 2:**
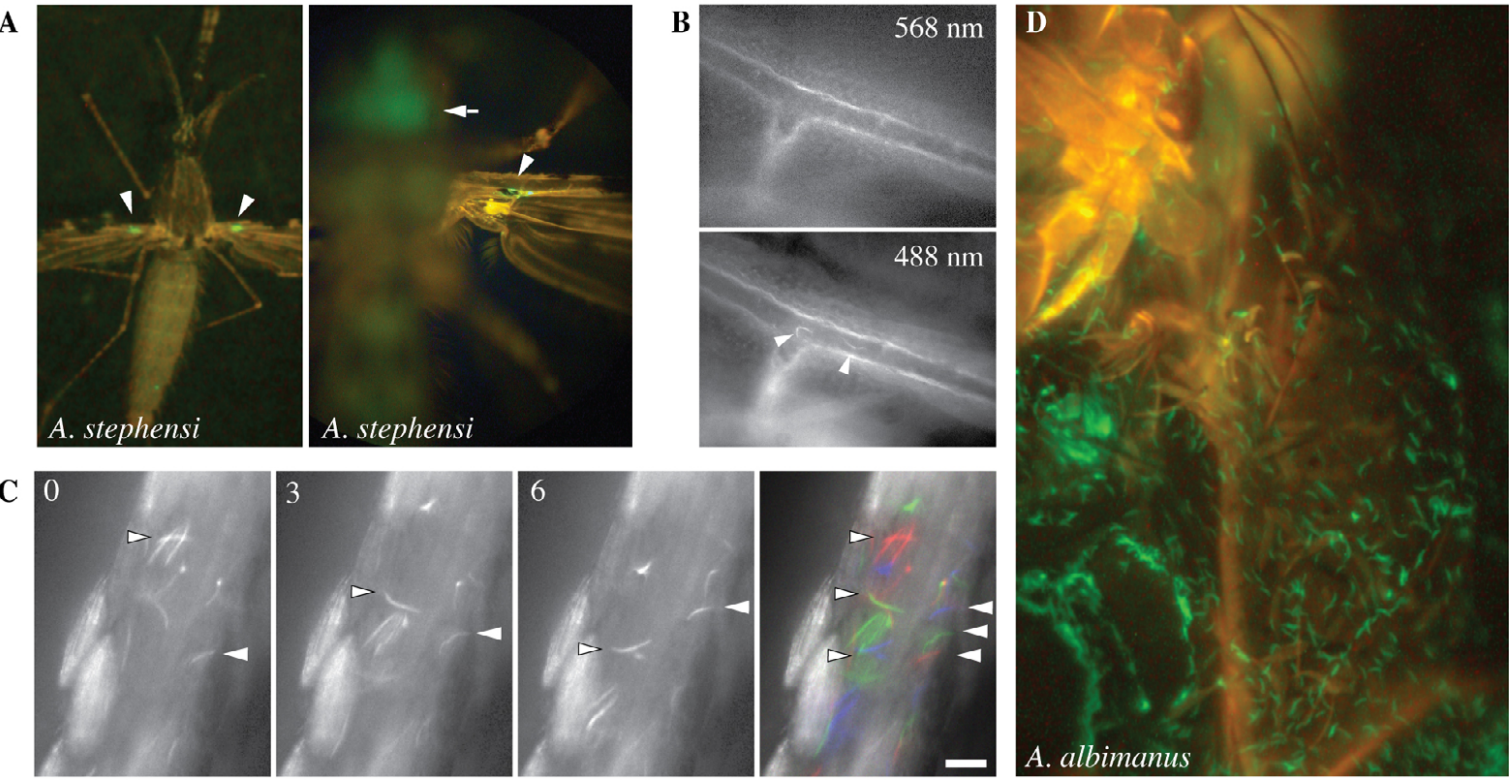
*In vivo *imaging of sporozoites in the haemolymph. (A) Left panel: An *A. stephensi *mosquito immobilized on a glass-slide for microscopy observation. Note the fluorescent signal at the base of the wings indicating haemolymph sporozoites (arrowhead). Right panel: An enlarged view of an *A. stephensi *mosquito viewed from the abdominal side indicating the fluorescent signal from sporozoites in the salivary gland (arrow) and from sporozoites in the veins of the wing (arrowhead). (B) Detection of individual sporozoites in the haemolymph. A vein of the wing imaged with a red filter (568 nm excitation) shows the auto-fluorescent mosquito tissue. The same region imaged with 488 nm excitation light shows the specific green fluorescence of the sporozoites (arrows) as well as the auto-fluorescent tissue. See also movie 1. (C) Three time-lapse images taken 3 seconds apart show the passive movement of sporozoites within the haemolymph of the mosquito tibia. The color image represents three images taken 3 seconds apart, pseudo-colored and overlayed to illustrate the movement of the sporozoites (see also movie 2). (D) Unusually many sporozoites (green) in the haemolymph of an *A. albimanus *thorax at 26 days post infection.

Close examination by epi-fluorescence microscopy of immobilized *A. albimanus *allowed the occasional detection of sporozoites within the haemolymph. Only a few *A. albimanus *salivary glands were found that contained sporozoites, usually in very small numbers (less than 10). In contrast, in *A. stephensi *mosquitoes, numerous sporozoites were readily detected in the haemolymph from as early as day 11 after infection (Figure 2a, b). Time-lapse analysis of immobilized *A. stephensi *mosquitoes showed that the sporozoites were passively flowing within the haemolymph (Figure 2c and Additional file 1 and 2, see also [23]). Sporozoites were found in any part of the body that was bathed by the haemolymph including the palps, the labium, the antennae, the legs, wings, abdomen and thorax (Figure 2d, Additional file 1 and 2). Examination of several thousand haemolymph sporozoites did not yield any evidence of active gliding motility by these sporozoites. It is, therefore, concluded that, at least in the *A. stephensi *– *P. berghei *system, sporozoites are passively transported by the haemolymph and eventually attach to the salivary glands and that very few *P. berghei *sporozoites are found in *A. albimanus*.

It had been previously shown that the efficiency of *P. falciparum*, *P. yoelii *and *P. berghei *development in *A. stephensi *can be increased by adding para-aminobenzoic acid (PABA) to the sugar water prior to the infectious blood meal [24,25]. It is also known that, as the presence of bacteria in the midgut of mosquitoes inhibits the infectivity of *P. falciparum *to *A. gambiae*, *A. stephensi *and *A. albimanus*, *Plasmodium *infection rates can be increased by adding antibiotics [25-27]. Therefore, in attempts to increase the efficiency of *P. berghei *development in *A. albimanus*, the sucrose solution was supplemented with 0.5 g/l PABA and 0.1 g/l penicillin/streptomycin (Pen/Strep). In this case, a higher number of *A. albimanus *females were infected by *P. berghei *and a higher number of oocysts per mosquito gut was found (Table 1). While in the absence of PABA and Pen/Strep, rarely more than one mosquito in a cage of 200 females was seen that had more than five oocysts, infection rates exceeding 20% were regularly achieved in the presence of supplements (Table 1). Of these infected mosquitoes, over 49% had more than five oocysts, while 22% showed more than 20 (Table 1).

**Figure 3:**
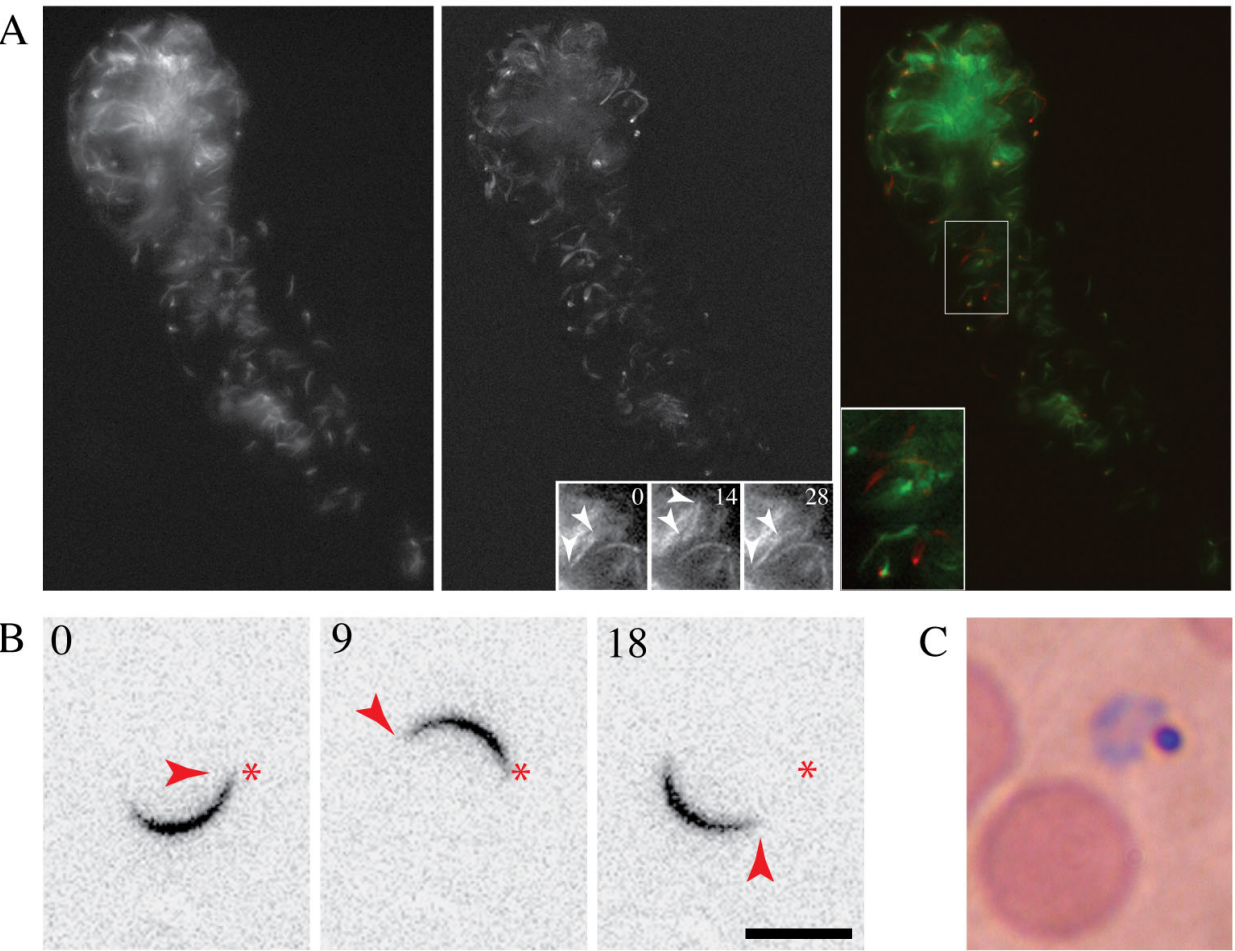
Infectious sporozoites in *A. albimanus*. (A) Isolated salivary gland of an infected *A. albimanus *mosquito at 26 days post infection. Sporozoites are shown in the left panel, while the movement occurring over 200 seconds is shown in the middle panel. This panel represents the projected standard deviation between time frames of a movie that spans 200 seconds with one image taken every 2 seconds. The insets show a typical back-and-forth moving sporozoite (arrowheads indicate ends of sporozoite). The time between frames is indicated in seconds. The right panel shows a merge of the static sporozoites (green) and the movement (red). See also movie 3. (B) An isolated sporozoite glides on a glass surface in the presence of 5% foetal calf serum. The asterix indicates the apical end of the sporozoites at 0 seconds, while the arrowhead indicates the apical end at the respective time frame. Time between frames is indicated in seconds. Scale bar: 10 μm. (C) Blood smear at 13 days after the injection of 2.000 salivary gland sporozoites shows an early trophozoite.

A closer examination revealed that *P. berghei *oocysts developed over a longer period of time (between 5 and 10 days delay) in *A. albimanus *than in *A. stephensi *midguts (Figure 1), which resulted in a delayed detection of fluorescent sporozoites in the haemolymph of *A. albimanus*. In virtually all *A. albimanus *mosquitoes with at least five oocysts some sporozoites could be detected in their haemolymph (Figure 2d). Nonetheless, the number of sporozoites within the salivary glands of these mosquitoes was usually very low. Occasionally, however, salivary glands were found that were infected by several hundred and sometimes several thousand sporozoites (Figure 3a).

To analyse *P. berghei *sporozoites in the *A. albimanus *salivary glands, the latter were incubated in cell culture medium containing foetal calf serum and visualized by time-lapse microscopy. Sporozoites within the glands were able to move (Figure 3a). Their main movement pattern was the "back-and-forth" type of motility previously observed for *P. berghei *sporozoites in *A. stephensi *salivary glands, in the absence or presence of serum [11]. Next, it was investigated if the sporozoites would be able to glide on a solid substrate, a prerequisite for infectivity to the mammalian host [28]. When incubated in medium containing foetal calf serum, *P. berghei *sporozoites isolated from infected *A. albimanus *glands moved on glass slides in a manner indistinguishable from *P. berghei *sporozoites isolated from infected *A. stephensi *glands (Figure 3b).

Next, the infectivity to the mammalian host of sporozoites isolated from *A. albimanus *salivary glands was investigated. Injection of 2,000 such sporozoites into the skin of mice caused red blood cell infection as determined by blood smear analysis (Figure 3c). This showed that *P. berghei *sporozoites, isolated from infected *A. albimanus*, were capable of invading both mosquito and mammalian tissues and to differentiate into red blood cell invading forms. However, in two separate experiments, intra-dermal injection of 20,000 sporozoites obtained from the haemolymph of *A. albimanus *mosquitoes failed to induce infection. Mice remained uninfected, which confirms that *Plasmodium *sporozoites undergo a maturation process in the salivary glands of *Anopheles *[29]. Qualitatively similar results were obtained by an infection of *A. albimanus *with *P. berghei *ANKA parasites expressing the green fluorescent protein [30].

Finally, whether *P. berghei *sporozoites can be transmitted to the mammalian host by the natural bite of *A. albimanus *mosquitoes during the third week post infection, was tested when sporozoites were present in the salivary glands of *A. albimanus*. Mouse infection was never induced even when over 10 infected mosquitoes were allowed to bite. Additionally, when artificial salivation was induced in immobilized *A. albimanus*, no ejection of sporozoites through the proboscis of the mosquitoes could be detected. Whether this reflects a true natural barrier to sporozoites inside *A. albimanus *or just the small number of sporozoites within the salivary glands remains to be determined. During previous studies using *P. berghei *infections of *A. stephensi*, sporozoites were already being ejected at day 11 post infection, when mosquitoes were artificially stimulated to salivate [11]. However, their numbers were very low (less than five in less than 20% of mosquitoes) and sporozoites were never observed within the first minutes during salivation. As those sporozoites ejected early during salivation are likely to be deposited in the skin [11,13], it is not surprising that even the combined bites of hundred *A. stephensi *mosquitoes at day 11 after the infectious blood meal were unable to infect mice. A similar situation might occur during infections with *A. albimanus*, where only very few sporozoites are taking up residence in the salivary glands and none was observed in the narrow parts of the salivary ducts.

While the difference between the findings described here and those of Vaughan et al. [17] might be due to the different strains of mosquitoes (A-2) and parasites (ANKA) used, it is more likely that the low number of oocysts developing in the absence of supplements escaped detection in the earlier study. Indeed, no difference was seen between fluorescent NK65 and ANKA strains in *A. albimanus*. This shows the advantage of using fluorescent parasites, as a single oocyst can be detected by careful observation in living mosquitoes and easily in dissected midguts.

## Conclusion

The feasibility to screen mosquito species for susceptibility to a malaria parasite species, using fluorescent parasites was demonstrated. Using this methodology, it was found that the rodent malaria parasite *P. berghei *is able to infect the major South American malaria vector *A. albimanus *and to develop into infectious sporozoites albeit at low frequency. This suggests that it should be possible to isolate *A. albimanus *lines that are highly susceptible to *P. berghei *infection and that such lines may provide useful new tools for studying the interaction between a model malaria parasite and a major malaria-transmitting mosquito.

## Authors' contributions

FF designed the study, FF and BM performed the experiments, FF, BM, IT, CB and RM discussed the experiments, FF, BM and RM analysed the data, IT and CB contributed the mosquitoes, FF and RM wrote the paper.

## Supplementary Material

Additional File 1 **Movie 1**. Sporozoites in the haemolymph of the mosquito wing. Two sporozoites are stuck in a vein of an *Anopheles stephensi *wing. Other sporozoites pass by with the flow of the haemolymph. 5 frames per second, movie length: 8 seconds. For best viewing loop the movie. Size: 4.4 MB. Click here for file

Additional File 2 **Movie 2**. Sporozoites in the hemolyph of an *A. stephensi *tibia. A large number of sporozoites float with the haemolymph in both directions. 1 frame per second, movie length: 74 seconds. Size: 3.3 MB. Click here for file

Additional File 3 **Movie 3**. Sporozoites in the salivary gland of an *Anopheles albimanus *mosquito. The gland was carefully isolated from an infected mosquito and placed in RPMI containing 5% foetal calf serum in a glass-bottom well dish. Note the displacement of sporozoites, mainly in a typical back-and-forth fashion. 0.1 frames per second, movie length: 100 seconds. For best viewing loop the movie. Size: 2 MB. Click here for file
